# Chemoprotective and chemosensitizing effects of apigenin on cancer therapy

**DOI:** 10.1186/s12935-021-02282-3

**Published:** 2021-10-29

**Authors:** Zahra Nozhat, Shabnam Heydarzadeh, Zahra Memariani, Amirhossein Ahmadi

**Affiliations:** 1 Institute of Smart Biomedical Materials, School of Materials Science and Engineering, Zhejiang Sci-Tech University, Hangzhou, 310018 China; 2 Cellular and Molecular Endocrine Research Center, Research Institute of Endocrine Sciences, Shahid Beheshti University of Medical Sciences, Tehran, Iran; 3 Department of Biochemistry, School of Biological Sciences, Falavarjan Branch Islamic Azad University, Isfahan, Iran; 4 Traditional Medicine and History of Medical Sciences Research Center, Health Research Institute, Babol University of Medical Sciences, Babol, Iran; 5 Pharmaceutical Sciences Research Center, Faculty of Pharmacy, Mazandaran University of Medical Sciences, Sari, Iran

**Keywords:** Apigenin, Chemoprotective, Chemosensitizing, Side effects, Molecular mechanisms

## Abstract

**Background:**

Therapeutic resistance to radiation and chemotherapy is one of the major obstacles in cancer treatment. Although synthetic radiosensitizers are pragmatic solution to enhance tumor sensitivity, they pose concerns of toxicity and non-specificity. In the last decades, scientists scrutinized novel plant-derived radiosensitizers and chemosensitizers, such as flavones, owing to their substantial physiological effects like low toxicity and non-mutagenic properties on the human cells. The combination therapy with apigenin is potential candidate in cancer therapeutics. This review explicates the combinatorial strategies involving apigenin to overcome drug resistance and boost the anti-cancer properties.

**Methods:**

We selected full-text English papers on international databases like PubMed, Web of Science, Google Scholar, Scopus, and ScienceDirect from 1972 up to 2020. The keywords included in the search were: Apigenin, Chemoprotective, Chemosensitizing, Side Effects, and Molecular Mechanisms.

**Results:**

In this review, we focused on combination therapy, particularly with apigenin augmenting the anti-cancer effects of chemo drugs on tumor cells, reduce their side effects, subdue drug resistance, and protect healthy cells. The reviewed research data implies that these co-therapies exhibited a synergistic effect on various cancer cells, where apigenin sensitized the chemo drug through different pathways including a significant reduction in overexpressed genes, AKT phosphorylation, NFκB, inhibition of Nrf2, overexpression of caspases, up-regulation of p53 and MAPK, compared to the monotherapies. Meanwhile, contrary to the chemo drugs alone, combined treatments significantly induced apoptosis in the treated cells.

**Conclusion:**

Briefly, our analysis proposed that the combination therapies with apigenin could suppress the unwanted toxicity of chemotherapeutic agents. It is believed that these expedient results may pave the path for the development of drugs with a high therapeutic index. Nevertheless, human clinical trials are a prerequisite to consider the potential use of apigenin in the prevention and treatment of various cancers. Conclusively, the clinical trials to comprehend the role of apigenin as a chemoprotective agent are still in infancy.

**Graphical Abstract:**

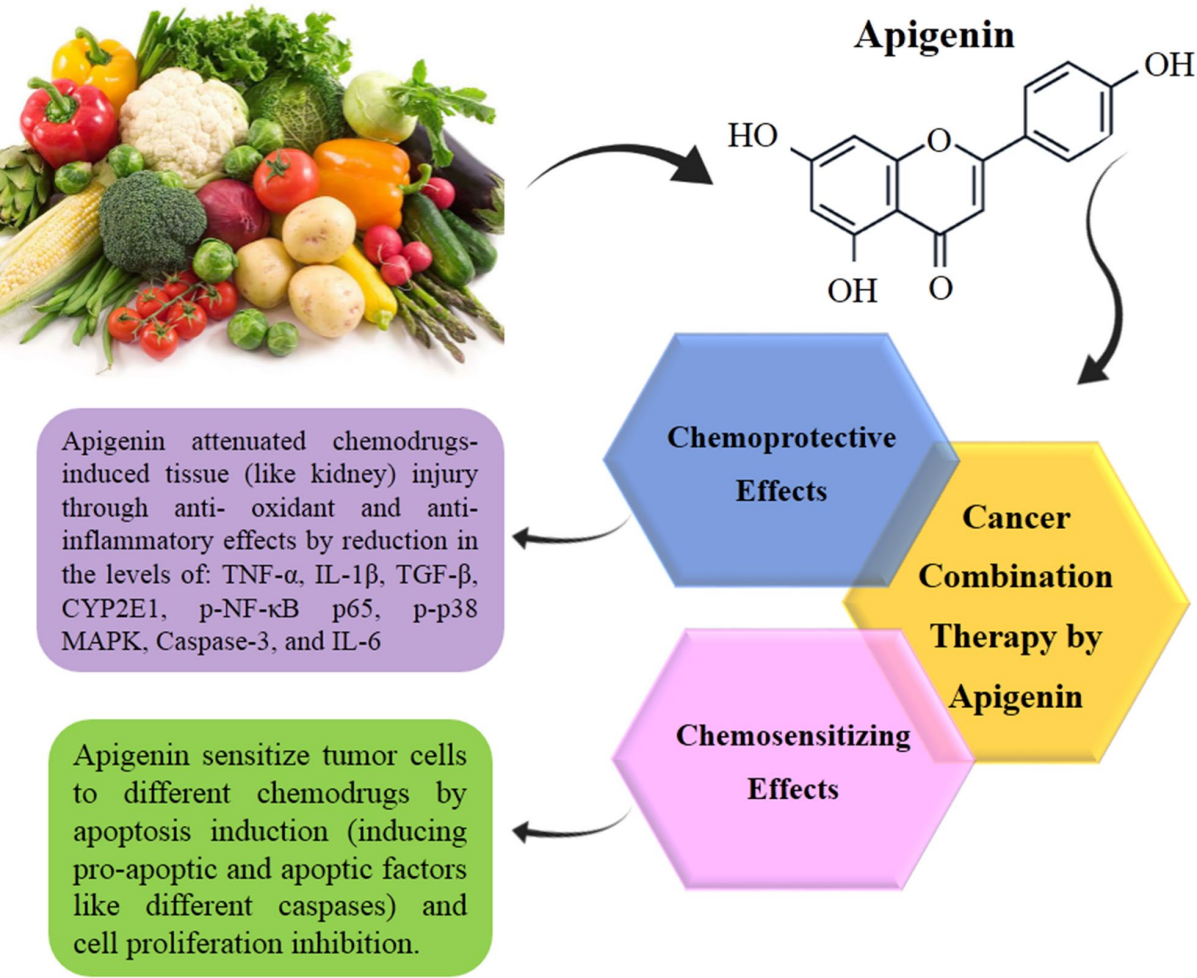

## Introduction

Combination therapy is promising to enhance the efficacy of cancer treatment and cope with the multiple genetic alterations in different cancer cells. It involves simultaneous administration of more than one type of treatments such as two or more chemotherapies or merging chemotherapy with radiation/adjuvant therapy. Sometimes, one or more natural anti-neoplastic products; herbal or fungal in origin with low molecular weight, may be used in combination therapy. Combinatorial therapy may be applied to cancer cell cultures, animal xenograft models, or clinical trials in cancer patients. In Biosystems, the combination of two drugs may exhibit synergistic, antagonistic, or additive effects compared to their properties in monotherapy. These approaches are attributed to minimize adverse effects of monotherapies; for instance, chemo drug resistance, low potency, diminishment of final doses along with their biological effects, and other side effects leading to patient’s death. However, radiosensitizing agents augment the radioresponsiveness of cancer cells making the therapy effective [1].

Plant-derived bioactive compounds are plausible novel therapeutic agents for the prevention of different types of cancers owing to a wide range of pharmacological effects [2]. The possible protective role of herbal extracts against drug-induced genotoxicity has been reported in some studies [3, 4]. Recently, apigenin has been introduced as an effective anti-neoplastic natural product. Nonetheless, it shows a moderate anti-cancer activity when administrated alone in its recommended human physiological dosages [5]. However, apigenin may perform synergistically by different cell signaling pathways, including down-regulation/up-regulation of transcription factors, activate/deactivate membrane receptors, cell signaling regulatory components, and apoptosis cascades. Combined treatment by apigenin may concurrently trigger diverse cell signaling pathways for sensitizing malignant tumor cells to chemo drugs [1]. Several studies [6–8] demonstrate that combination therapy with apigenin in different types of cancers, not only enhances the efficacy of chemotherapy but also reduces the side effects by targeting multiple cell signaling pathways.

This review summarizes recent advances in combinatorial therapy, particularly demonstrating the synergistic effects of apigenin on various cancer cell cultures and animal models. Since chemoresistance is one of the main hindrances in cancer therapy, we aimed to describe a natural bioflavonoid as apigenin, based on its chemosensitizing effect. Many tumors are initially chemo drug responsive but eventually develop drug resistance. Apigenin is reported to be useful in combination therapies along with its role as a chemosensitizer on chemo drugs. Table 1 abridges the combination of apigenin with several chemo drugs. Apigenin can make tumor cells vulnerable to chemotherapeutic agents, so conventional chemo drugs can be amalgamated with flavonoids, giving a new direction to chemotherapy. We comprehensively presented nearly all important research findings on apigenin as an important co-treatment drug in cancer therapy. Nevertheless, we lack data for clinical trials of apigenin in combinatorial therapy and its significance as chemoprotective and chemosensitizing agent. We strongly recommend perform further studies on this subject.

**Table 1:** Apigenin combination therapy with different types of chemotherapy agents

Chemotherapy agent	FDA approve of chemodrug	Cancer type	Sample type	Dose of chemodrug	Dose of apigenin	Effects of combined treatment	Target Molecules	Refs.
5-Fluorouracil	Approved in 2000	Human breast cancer	MDA-MB-453 cell line	90 µM	> 10 µM	More inhibition of the cell proliferation and more induction of the apoptosis compared with the chemodrug alone	↓ ErbB-2 ↓ AKT ↑ Caspase-3	[44]
		Head and neck squamous cell carcinoma	SCC25 cell line	10–100 µM	5 µM	More inhibition of the cell proliferation compared with the chemodrug alone	NA	[51]
		Human pancreatic cancer	BxPC-3 cell line	50 µM	13 µM	More inhibition of the cell proliferation compared with the chemodrug alone	NA	[52]
		Hepatocellular carcinoma	SK-Hep-1 and BEL-7402 cell lines and HCC xenograft model	100 μg/ml for cell lines treatment and 20 mg/kg for in vivo treatment	4 μmol/L for cell lines treatment and 20 mg/kg for in vivo treatment	In HCC cells significantly enhanced the cytotoxicity of 5-FU and increased levels of ROS and a decrease in the mitochondrial membrane potential In vivo, combined treatment significantly inhibited the growth of HCC xenograft tumors	↑ Caspase-3 ↑ PARP ↓ Bcl-2	[53]
		Induced oral mucositis by 5-FU as the common side effect of 5-FU	Syrian hamster model	60 mg/kg body weight	40 mg/kg	Enhancement of the healing of oral mucositis induced by 5-fluorouracil	NA	[55]
		Solid Ehrlich carcinoma	Swiss albino male mice	20 mg/kg	100 mg/kg	Significant decrease in tumor volume, tissue glutathione peroxidase and total antioxidant capacity and alleviated the histopathological changes with significant decrease of Ki-67 proliferation index compared to vehicle treated SEC control group	↑ Beclin-1 ↑ Caspases 3, 9 ↑ JNK ↓ Mcl-1	[56]
		Human leukemia	TIB-152, TIB- 202, CCL-119 and CCL-243 cell lines	0.01–10 µM	10 µM	Synergistic decrease in ATP levels, induction of cell-cycle arrest and apoptosis	↑ Caspase-3 ↓ ATP	[3]
		Human colorectal cancer	HCT-15 and HT-29cell lines and CRC xenograft model	5 mM	7.5 mM	Enhanced anti-proliferative effect Induction of cellular arrest and/or apoptosis Inhibition of angiogenesis	↑AMPK ↑ ROS ↓ COX-2 ↓ HIF-1α	[61]
Cetuximab	Approved in 2004	Head and neck squamous cell carcinoma	Cal27 and LICR-HN1 cell lines	15 nM	17.5 mM	Reduced the cells survival	NA	[66]
		Head and neck squamous cell carcinoma	LICR-HN5 R9.1 and SC263 10.2 cell lines	15 nM	25 mM	Inhibited cell proliferation	↑ S100A8 ↑ PLAU in SC263 10.2 ↓ PLAU in LICR-HN5 R9.1 ↓ CST6 ↓ FOSL1 ↓ VIM	[68]
		Human nasopharyngeal carcinoma	HONE1 and CNE2 cell lines, NPC nude mice	0.25 mg/ml for cell lines treatment 50 mg/kg/day for in vivo treatment	50 µM for cell lines treatment 0.8 mg/mouse/day for in vivo treatment	Increased the anti-tumor ability of cetuximab Enhanced the effect of cetuximab on the induction of apoptosis and cell cycle arrest Elevated the ability of cetuximab to inhibit the EGFR signaling pathway	↓ p-EGFR ↓ p-AKT ↓ p-STAT3 ↓ Cyclin D1	[75]
Cisplatin	Approved in 1978	Murine melanoma	B16-BL6 cell line	2 mg/kg	25 mg/kg	More inhibition of the cell proliferation compared with the chemodrug alone	NA	[81]
		Human laryngeal carcinoma	Hep-2 cell line	5 µg/ml	10, 40, 160 µM	Apigenin enhanced the cisplatin-induced suppression of Hep-2 cell growth	↓ GLUT-1 ↓ p-AKT	[82]
		Human normal renal cells	Immortalized human renal proximal tubular epithelial (HK-2) cells	40 µM	5, 10, 20 µM	Apigenin improved cisplatin-induced apoptosis	↓ caspase-3 ↑ PARP ↓ P53 ↑ PI3K/AKT	[86]
		Human cervical, alveolar basal epithelial, colon, non-small cell lung, and breast cancers	HeLa, A549, HCT 116, H1299, and MCF-7 cell lines	2.5, 5, 10 µM	30 µM	Apigenin enhances the cytotoxic effect of cisplatin and cisplatin-induced apoptosis	↑ MAPK ↑ P53	[87]
		Male BALB/c mice	Kidney tissue	20 mg/kg	5, 10, 20 mg/kg	Apigenin attenuated cisplatin-induced kidney injury through anti- oxidant and anti-inflammatory effects	↓ TNF-α ↓ IL-1β ↓ TGF-β ↓ CYP2E1 ↓ p-NF-κB p65 ↓ p-p38 MAPK	[88]
		Female Wistar Albino mice	Kidney tissue	7.5 mg/kg	3 mg/kg	Apigenin attenuated cisplatin-induced kidney injury through anti- oxidant and anti-inflammatory effects	↓ caspase-3 ↓ TNF-a ↓ IL-6	[90]
		Human breast cancer	MDA-MB-231 and HCC1806 cell lines	6 μg/ml for MDA-MB-231 and 12 μg/ml for HCC1806	14 μg/ml for MDA-MB-231 and 8 μg/ml for HCC1806	Induction of cell apoptosis	↓ hTERT ↓ Hsp90 ↓ p23	[91]
		Human prostate cancer	PC3 cell line	15 mM	7.5 mM	Induction of the cell cycle arrest	↓ p-PI3K ↓ p-Akt ↓ NF-kB ↑ p21 ↑ CDK-2 ↑ CDK-4 ↑ CDK-6	[92]
		Human ovarian adenocarcinoma	SKOV-3 and SKOV-3/DDP cell lines	2 µM	50 µM	Induction of the cell cycle arrest and apoptosis promotion	↓ cyclin D ↓ cyclin B ↓ cyclin E ↓ Bcl-2 ↓ Mcl-1 ↑ Caspase-3 activity	[93]
Cyclophosphamide	Approved in 1959	Human leukaemia	Jurkat CCRF-CEM THP-1 KG-1a cell lines	10 µM	10–50 µM	Induction of the cell cycle arrest and apoptosis promotion	↑ Caspase-3 activity ↑ Caspase-8 activity ↑ Caspase-9 activity ↑ γH2AX foci ↓ ATP	[99]
Doxorubicine	Approved in 1974	Human hepatocellular carcinoma	BEL-7402/ADM and EL-7402 cell line	10 µM	10 µM	Induction of the cell cycle arrest and apoptosis promotion	↓ Nrf2 ↓ PI3K/AKT	[101]
		Human hepatocellular carcinoma	BEL-7402/ADM cell line	8 µg/ml	10 µM	Induction of the cell cycle arrest and apoptosis promotion	↓ Nrf2 ↑ miR-101	[102]
		Human hepatocellular carcinoma	BEL-7402/ADM cell line	2 µM	10 µM	Induction of the cell cycle arrest and apoptosis promotion	↓ ATG7 ↑ miR-520b	[103]
		Human prostate cancer	C4-2B and TaxR cell lines	20 nM	10 mM	Induction of the cell cycle arrest and apoptosis promotion	↓ ABCB1	[107]
		Human uterine sarcoma	MES-SA/Dx5	2–8 µM	10 µM	Induction of the cell cycle arrest and apoptosis promotion	↓ ABCB1	[109]
		Human breast cancer	MCF-7/ADR cell line	20 µg/ml	100 µM	Induction of the cell cycle arrest and apoptosis promotion	↓ ABCB1 ↓ STAT3	[110]
		Human leukaemia	Jurkat CCRF-CEM THP-1 KG-1a cell lines	10 µM	10–50 µM	Induction of the cell cycle arrest and apoptosis promotion	↑ Caspase-3 activity ↑ Caspase-8 activity ↑ Caspase-9 activity ↑ γH2AX foci ↓ ATP	[111]
		Human hepatocellular carcinoma	HepG2 cell line	1 µM	50 µM	Glycolysis inhibition	↓ HK2 ↓ LDHA	[114]
		Human ovarian adenocarcinoma	SKOV-3 cell line	0.5 µM	26 µM	Induction of the cell cycle arrest and apoptosis promotion	↑ Caspase-9 activity ↑ COX-2 ↑ Bcl-2	[115]
Gemcitabine	Approved in 1996	Human pancreatic cancer	BxPC-3 cell line	10 µM	13 µM	More inhibition of the cell proliferation compared with the chemodrug alone	NA	[52]
		Human pancreatic cancer	MiaPaCa-2 and AsPC-1 cell lines	10 µM	25 or 50 µM	Induction of the cell cycle arrest and apoptosis promotion	↓ NFκB ↓ AKT	[118]
		Human pancreatic cancer	CD18 and AsPC-1 cell lines	10 µM	25 µM	Induction of the cell cycle arrest and apoptosis promotion	↓ AKT	[119]
Navitoclax	Not approved	Human colon cancer	DLD1, HCT116, HCT-8, HT29 and SW48 cell lines	1 µM	20 µM	Induction of apoptosis	↑ Mcl-1 ↑ Bim ↑ Bax ↓ AKT ↓ ERK	[122]
		EGFRm tumors	H1975, HCC827, H1650, H3255, SK-MEL-28 cell lines	2 µM	15 µM	Inhibition of cell growth and proliferation	↓ EGFR ↑ Noxa ↓ AKT ↓ FOXO3a	[123]
Paclitaxel	Approved in 2002	Human cervical epithelial, lung epithelial, and negroid hepatocyte carcinomas	HeLa, A549 and Hep3B cell lines	4 nM	15 µM	Induction of apoptosis	↓ SOD ↑ Caspase-2 activity	[127]
		Human hepatocellular carcinoma	HepG2 cell line	6.5, 25, 100 nM	40 µM	Reverse hypoxia-induced drug resistance	↓ HIF-1a ↓ AKT ↓ HSP90	[128]
		Human ovarian adenocarcinoma	SKOV-3 cell line	5 nM	10 µM	Induction of the cell cycle arrest and apoptosis promotion	↓ SOD ↑ ROS ↑ Caspase-3 activity ↑ Bax ↓ Bcl-2	[130]
Sorafenib	Approved in 2005	Human hepatocellular carcinoma	HepG2 cell line	5 μM	50 μM	Induction of the cell cycle arrest and apoptosis promotion	↑ caspase-3 ↑ caspase-8 ↑ caspase-10 ↑ BID ↑ p21 ↑ p16	[132]
Tamoxifen	Approved in 2000	Human breast cancer	Xenograft female albino Wistar rats	50 mg/kg	100 and 200 mg/kg	Increased the activity of anti-oxidant enzymes and reduced angiogenesis	↑ SOD ↑ GPx ↑ CAT ↓VEGF	[134]
		Human breast cancer	MCF-7 cell line	100 nM	10 μM	Inhibition of cell growth	↓ ERα ↓ AIB1	[135]
Abivertinib	Not approved	Human DLBCL lymphoma	U2932 and OCI-LY10 cell lines and DLBCL xenograft BALB/c nude mice model	156 nM for cell lines and 30 mg/kg for xenograft model	2.5 μM for cell lines and 2 mg/kg for xenograft model	Induction of the cell cycle arrest and apoptosis promotion	↓ BCL-XL ↓ PI3K/mTOR ↓ p-GS3K-β ↓ BTK ↑ caspase-3 ↑ caspase-8	[137]
Apo2L-TRAIL	Not approved	Human prostate cancer	DU145 and LNCaP cell lines	50 ng/ml	20 μM	Induction of apoptosis	↓ ANT2 ↑ DR5	[139]
Chlorambucil	Not approved	Human leukemia	Jurkat CCRF-CEM THP-1 KG-1a cell lines	0.01–10 µM	50 µM	Synergistic decrease in ATP levels, induction of cell-cycle arrest and apoptosis	↑ Caspase-3 activity ↑ Caspase-8 activity ↑ Caspase-9 activity ↑ γH2AX foci ↓ ATP	[99]
Gefitinib	Approved in 2003	Human NSCLC	NCI-H1975 and 95-D cell lines	40 µM	40 µM	Induction of the cell cycle arrest and apoptosis promotion	↑ Caspase-3 activity ↑ PARP-1 ↓ Bcl-2 ↑ BIM ↑ Bax ↓ p-AMPK-α	[141]
IFN-γ	Approved in 1991	Human cervical cancer	HeLA cell lines	100 ng/ml	10 µM	Induction of the cell cycle arrest and apoptosis promotion	NA	[130]

The study explains combinatorial strategies to overcome drug resistance and amplification of anti-cancer properties in different types of cancers. Herein, the co-administration of apigenin with the chemo drugs such as 5-Fluorouracil, Cetuximab, Cisplatin, Cyclophosphamide, Doxorubicin, Gemcitabine, Paclitaxel, Sorafenib, Tamoxifen, Abivertinib, Apo2L-TRAIL, Chlorambucil, Gefitinib, Interferon-gamma, Methotrexate, and Vincristine is presented.

## Different forms of apigenin and its bioavailability

**Fig. 1:**
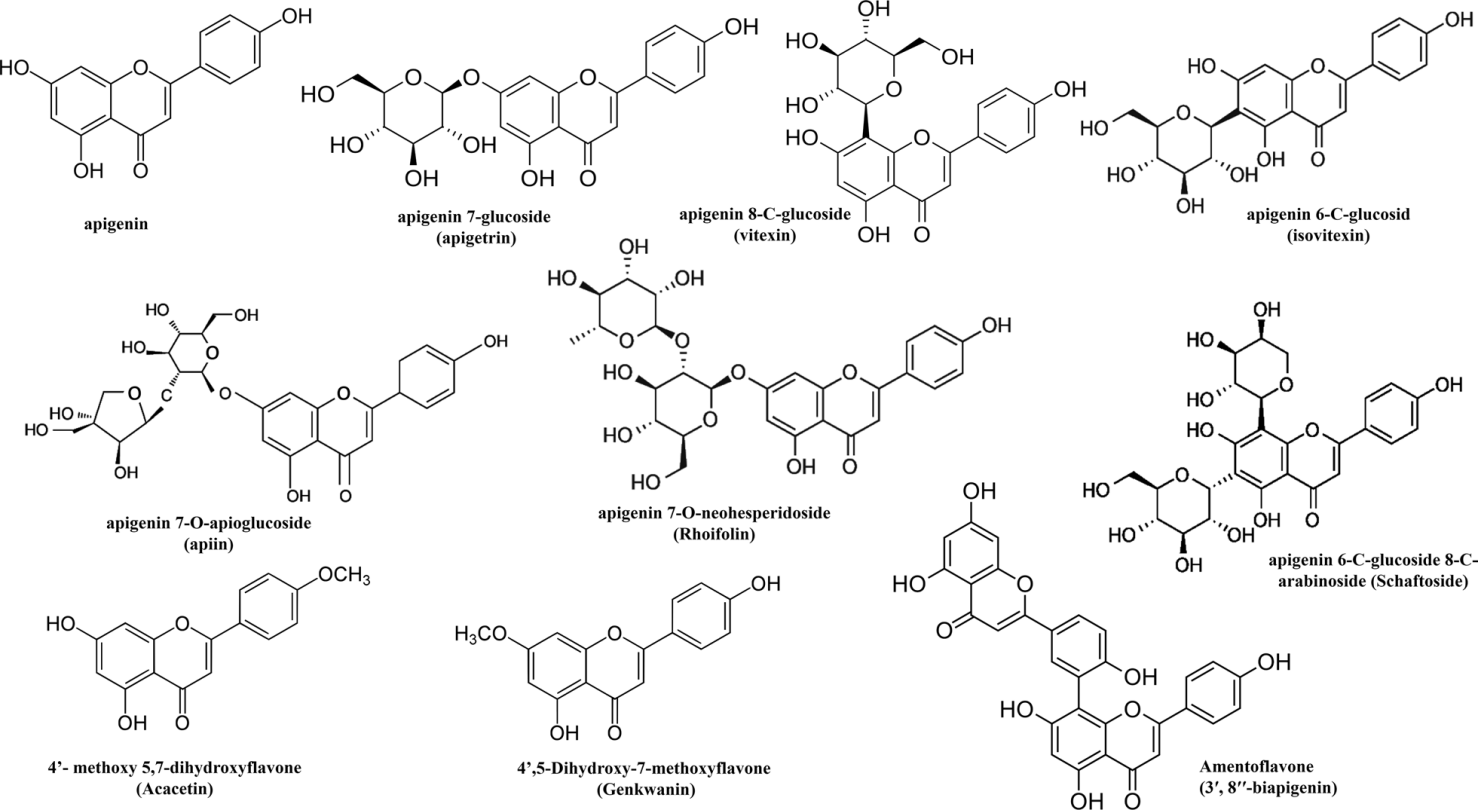
Chemical Structure of different forms of apigenin

Apigenin is a well-known flavonoid with a flavone structure based on the backbone of 2-phenylchromen-4-one (2-phenyl-1-benzopyran-4-one). It is a trihydroxyflavone that has hydroxyl groups at positions 4', 5, and 7 [9]. Apigenin mostly occurs as a glycoside found in many fruits, vegetables, medicinal plants, and occasionally in as aglycone. Various factors, such as environmental growth condition, genetics, and developmental stage etc., determine the form of apigenin glycoside present in plants [10]. Some of the naturally occurring apigenin glycosides include apigenin 7-glucoside (apigetrin), apigenin 8-C-glucoside (vitexin), apigenin 6-C-glucoside (isovitexin), apigenin 7-*O*-apioglucoside (apiin), apigenin 7-*O*-neohesperidoside (Rhoifolin), apigenin 6-C-glucoside 8-C-arabinoside (Schaftoside), 4'-methoxy 5,7-dihydroxyflavone (Acacetin), and 4',5-Dihydroxy-7-methoxyflavone (Genkwanin) [11]. Moreover, in some plants, apigenin dimer is also found e.g. Amentoflavone (3′, 8′′-biapigenin) with pharmacological activities found in *Hypericum perforatum* L. [12, 13], and *Ginkgo biloba* L. [14]. Chemical structures of various forms of apigenin are represented in Fig. 1. Apigenin aglycone or its conjugated forms exist in dietary plants (such as fresh parsley, celery seed, dried oregano, and cilantro) and medicinal herbs. Dried parsley and Chamomile tea have a particularly high amount of apigenin among vegetables or herbs [15]. The amount of apigenin as a flavonoid in the daily diet (mg/100 g of edible portion) may vary in different fruits and vegetables. It has been stated that fresh parsley and salary contain 215.46 mg/100 g FW and 24.02 mg/100 g of apigenin respectively. Apigenin also occurs in spices from the family Lamiaceae such as thyme, oregano, mint, rosemary, and sage [16]. C-glycosides is reported as a dietary supplement in algae [17].

Many research groups have developed new compounds based on apigenin to improve its physicochemical properties and bioavailability for clinical trials, because like many other flavonoids, apigenin has poor solubility in water, moderate solubility in alcohol, and low metabolic stability [18]. Based on the number of substituents in their molecules, flavonoids have different levels of decomposition. For example, hydroxyl groups stimulate flavonoids degradation, while methoxyl groups and sugar moiety avert flavonoids decomposition during different extraction processes [19]. Glycoside forms of apigenin, like -7-*O*-glucoside and its acylated derivatives have more solubility in water [20], while β-glycosides of apigenin show the best bioavailability [21]. The novel approaches to improve the solubility and stability of apigenin include different delivery systems such as liposomes, polymeric micelles, nanosuspension, etc. Furthermore, apigenin, as an anti-cancer natural product may inhibit several cancer cell lines proliferation [22].

Generally, the C2–C3 double bond in flavonoids structure, including apigenin, is allied to the anti-cancer activity by inhibiting membrane efflux transporters in resistant breast cancer cells [23, 24]. The presence of the OH group in C-5 along with the O–CH3 group in C-3 is attributed to inhibitory activity against cancer resistance protein [25]. Furthermore, the study of a theoretical model of anti-cancer properties of flavonoids suggests that the 5,7-dimethoxy flavonoids are the most potent anti-cancer agents than others [26]. Substitutions of *p*-hydroxyl, 3, 5′-dimethoxy, 5′-amino, and 2′-chloro at ring B of flavone are vital for anti-cancer activity via various mechanisms. Besides, substituted aliphatic/aromatic amino groups on C-6/C-8, on ring A hydroxyl and chloro groups at C-5 and C-6, respectively, and 3-methoxy on ring C are significant for the anti-cancer effect [27]. Alkyl amine moieties also escalate the anti-cancer activity of apigenin derivatives by enhancing their lipophilicity. Alkyl amine moieties linked to the apigenin ring system at C-7 impede proliferation in A549, HeLa, HepG2, and MCF-7 cancer cell lines [28]. Protoflavones; derived from apigenin with non-aromatic B-ring and OH group at C-1′, are potent anti-cancer agents in vitro and in vivo that prevent multidrug resistance by evading P-glycoprotein both [29]. Moreover, the synthesized triazolyl analogs of apigenin have been considered to induce apoptosis in SKOV3 ovarian cancer cells. Particularly, 1, 2, 3-triazole analogs synthesized from apigenin-7-methyl ether possess stability against acidic/basic hydrolysis and facilitate the interaction with membrane proteins [30].

## Safety and toxicity of apigenin

Flavonoids are assumed to be safe nutritionally, while apigenin implicates low toxicity [31]. However, evaluation of the acute toxicity of apigenin resulted in no mortality or signs of toxicity in mice/rats at oral doses up to 5000 mg/kg [32]. Moreover, in vitro evaluation of carcinogenicity proved that apigenin has no toxic or mutagenic effects [33, 34]. Intriguingly, after 30 min treatment in vitro, the hemolytic activity of apigenin was reported to be lower than the acceptable limit of 5% signifying its potential safety in intravenous dosages [35]. Apigenin glycosides in dietary sources have not been reported to have any toxic effects, however, consumption in high doses or prolonged usage as dietary or pharmaceutical supplements must be considered. In male Swiss mice, apigenin stimulates oxidative stress followed by liver damage at doses of 100 and 200 mg/kg, when administered intra-peritoneal as a single dose [36]. Correspondingly, in an in vitro study, the treatment of a normal trout liver cell line with apigenin for 24 h caused hydroxylation that might hamper the cell growth at 25 μM concentration [37].

## Pharmacokinetics of apigenin

Pharmacokinetics determines the amount of consumed apigenin available to the human gut microbiota. The metabolism and conjugation of apigenin occurs in the gastrointestinal tract before entering into the systemic circulation. As a dietary flavonoid, the absorption and excretion of apigenin were investigated clinically by ingesting a bolus of 2 g parsley (containing 65.8 ± 15.5 µmol apigenin) in healthy subjects. Analysis of urine, plasma, and RBCs revealed that the concentration of apigenin in the blood of all participants first increased; after a bolus ingestion, and then decreased in 28 h below the detection limit. The concentration of apigenin in 24-h urine samples was 0.22% of the ingested dose [38]. In another clinical study regarding ingestion of parsley (3.73–4.49 mg apigenin/MJ), 0.58% of the apigenin was excreted in the urine [39]. Apigenin metabolism by gut microbiota can be detected in feces after oral ingestion. Oral administration of radiolabeled apigenin in rats was traced, where 51% retrieved in urine and 12% in feces during 10 days. It was specified that apigenin could be bioaccumulated due to its slow metabolism and elimination [40]. Apigenin, in its aglycone form, is quickly absorbed in the duodenum apart from its dietary consumption in a perfused rat intestinal model [41]. Similarly, apigenin glycosides are absorbed as it is from the stomach to the large intestine and deglycosylated in the cecum [42]. Oral administration of apigenin leads to low concentration in blood [43]. Furthermore, intravenous administration of the same amount of apigenin and its glycosides renders a higher concentration of apigenin in blood than its glycosides. The metabolism of absorbed apigenin in rat liver follows Phase I in the presence of NADPH, cytochrome P450, and enzymes like flavin-containing monooxygenase [44]. In both humans and rats, during phase II, apigenin mainly undergoes glucuronidation and sulfation [45]. Luteolin, such as glucuronide and sulfate conjugates are the main blood metabolites of apigenin [40]. Further glucuronidation of apigenin also occurs in the intestine [45].

## Combination therapies with apigenin

### 5-Fluorouracil (5-FU)

5-fluorouracil (5-FU) is a pyrimidine analogue with a potential inhibitory effect on thymidylate synthase (the role of thymidylate synthase is the generation of deoxythymidine monophosphate (dTMP) from deoxyuridine monophosphate (dUMP) in the folate cycle). Such inhibition causes the accumulation of dUMP, which in turn disrupts the folate cycle and eventually results in DNA damage and cell death [6]. 5-FU, as an FDA approved chemo drug in 2000, is applied for treatment of various types of cancers including colon cancer, esophageal cancer, stomach cancer, pancreatic cancer, breast cancer, cervical cancer [7], head, neck, and skin cancers [8, 46]. Although, 5-FU is a phenomenal chemotherapeutic agent but development of the drug resistance and cytotoxicity limit its clinical usage. Therefore, it seems that novel therapeutic strategies are required to modulate its cytotoxicity [7] and combination therapy with apigenin is one of the particular interests.

#### Human breast cancer

In 2009, Choi and Kim [47] investigated the effects of 5-FU in combination with apigenin on cell proliferation and apoptosis in human breast cancer. For this purpose, they selected the MDA-MB-453 cell line with overexpression of erythroblastic oncogene B-2 (ErbB-2, also as known human epidermal growth factor receptor 2 or HER-2/neu) compared to the other human breast cancer cell lines. The result turned out with a synergistic effect between the two compounds, when the cells were exposed to 5-FU and apigenin at 90 μM and 10 μM concentrations, respectively. This co-therapy led to a significant reduction in ErbB2 and protein kinase B (AKT) expression and AKT phosphorylation as compared to monotherapy [47]. Increased resistance to 5-FU in monotherapy has been attributed to the overexpression of ErbB2 in human breast cancer cells. Apigenin is reported to induce apoptosis by down-regulating the *ErbB-2* expression [48]. Treatment by 5-FU alone could not affect the expression of ErbB*-2* in MDA-MB-453 cells, while its co-administration with apigenin notably decreased the expression of ErbB2. Additionally, this combinatorial therapy significantly induced the apoptosis in the treated cells (up to 50%) compared with the induced apoptosis with 5-FU alone. It was shown that the combination of 5-FU and apigenin exerted apoptosis induction by the reduction in AKT expression and AKT phosphorylation [47]. Several studies demonstrated that anti-cancer effects of the apigenin were related to inhibition of the AKT pathway [49, 50]. Choi and Kim [47] presented that co-treatment with 5-FU and apigenin diminished AKT expression and phosphorylation in human MDA-MB-453 cells which could be an important mechanism of this combination therapy. Moreover, caspase-3; a major death protease catalyzing the cleavage of several key cellular proteins, was considered as an apoptosis biomarker in this study. According to the results, co-administration of 5-FU and apigenin exhibited an anti-cancer activity by reducing cell proliferation and stimulating apoptosis and the authors attributed this finding to the significant increase in the caspase-3 expression in the breast cancer cell line. Finally, the authors suggested that the obtained results could be encouraging for the clinical trials of co-treatment of 5-FU and apigenin in human breast cancers to curtail the limitations of 5-FU.

#### Head and neck squamous cell carcinoma

The experimental findings of Chan et al. [51] supported the chemopreventive role of apigenin against head and neck squamous cell carcinoma (HNSCC). In this study, it was proved that the cytotoxicity of 5-FU to HNSCC SCC25 cell line was enhanced by apigenin. To determine the effects of apigenin co-treatment with 5-FU, SCC25 cells were treated for 72 h with 5 μM apigenin and 2.5–200 μM 5-FU alone. In monotherapy, apigenin and 5-FU reduced the cell viability by approximately 20% and 0.8–74.3%, respectively. The co-administration of apigenin (5 μM) and 5-FU (10, 20, 50, and 100 μM) in SCC25 cells for 72 h increased the cytotoxicity of 5-FU in a dose-dependent manner, particularly when 5 μM apigenin was applied in the combination with 20 μM 5-FU. Consequently, apigenin alone up-regulated both TNF-R and TRAILR, down-regulated B-cell lymphoma-2 (Bcl-2), activated caspase-3, and induced apoptosis in the SCC25 cell line. The synergistic effects of apigenin and 5-FU on the SCC25 cell line have been ascribed to the difference between their underlying intracellular pathways alone and that of their combination therapy [51]. Although, the authors suggested that this co-treatment could be an effective novel strategy against HNSCC, they did not ponder the molecular mechanism of its action.

#### Pancreatic cancer

To investigate the chemosensitizing effects of apigenin on pancreatic cancer cells (BxPC-3), Johnson et al. [52] treated BxPC-3 cell line by 5-FU (50 μM) alone for 60 h leading to 59% inhibition in cell growth. Co-administration of 50 μM 5-FU and 13 μM apigenin causing 71% inhibition in cell growth. Corresponding to the previous studies [47, 51], they concluded that the combination therapy was more effective than apigenin and 5-FU alone. The authors elucidated that this finding could be a result of the potential competition between anti-oxidant and pro-oxidant activities of apigenin [52], yet they did not explain possible cell signaling pathways underlying simultaneous administration of apigenin and 5-FU.

#### Liver cancer

Hu et al. selected apigenin as a natural chemosensitizer to make hepatocellular carcinoma (HCC) cells more vulnerable to 5-FU [53]. For this purpose, they used SK-Hep-1 and BEL-7402 cell lines and an animal HCC xenograft model. The growth of SK-Hep-1 and BEL-7402 cells was significantly reduced by apigenin or 5-FU in a dose-dependent manner. The simultaneous treatment of 4 µmol/L apigenin significantly increased the cytotoxicity of 5-FU in 100 µg/ml concentration in both HCC cells lines compared with apigenin or 5-FU alone. In the HCC xenograft animal model, co-administration of apigenin (20 mg/kg, five times/week for 3 weeks) and 5-FU (20 mg/kg for 5 consecutive days) notably impeded the growth of the xenograft tumors. This Combinatorial treatment enhanced reactive oxygen species (ROS) and subsequently decreased the potential of the mitochondrial membrane (DΨm) in HCC cells. The ROS production in cells co-treated with apigenin and 5-FU was greater than in the cells treated with 5-FU alone and there was no significant difference in DΨm between the control and apigenin-treated cells. On the other hand, co-incubation of the cells in apigenin and 5-FU induced the apoptosis signaling pathways, by down-regulation of Bcl-2 expression, diminishment of DΨm, and up-regulation of caspase 3 and poly (ADP-ribose) polymerase (PARP). According to these results, apigenin may enhance the sensitivity of liver cancer cells to 5-FU by the activation of the mitochondrial apoptosis pathways. So, the authors concluded that apigenin could be a potential chemosensitizer for 5-FU and utilized as a novel co-therapies in liver cancer treatments [53].

#### Solid Ehrlich carcinoma

To investigate anti-cancer effects of combination therapy of apigenin and 5-FU, Gaballah et al. [54] considered solid Ehrlich carcinoma (SEC), a murine undifferentiated malignancy that is mammary in origin. In this study, 80 SEC mice were divided into 4 equal groups including: 1) SEC control group, 2) SEC treated by 5-FU, 3) SEC treated by apigenin, and 4) SEC treated by 5-FU plus apigenin. The co-administration of 5-FU and apigenin improved the survival rate in the groups receiving combination therapy as compared to the groups treated with 5-FU or apigenin alone [54]. Beclin-1 is another protein that regulates the autophagy [55] and its down-regulation has been observed in breast cancer [56]. In this study, Gaballah et al. [54] proposed that the co-administration of 5-FU and apigenin markedly increased Beclin-1 protein levels in SEC compared to 5-FU or apigenin treated groups. These findings could be helpful to reduce chemoresistance of SEC cells to 5-FU by induction of the cell autophagy. The activation of JNK can induce Bcl-2 phosphorylation, resulting in the release of Beclin-1and autophagy [57]. In general, these results revealed a pivotal role of JNK in apigenin-induced autophagy and apoptosis. The activation of the Mcl-1; an inhibitor of apoptosis pathways, may trigger carcinogenesis and develop chemo drug resistance in cancer cells [58]. In Gaballah's study, the down-regulation of Mcl-1 has been attributed to apigenin induced AKT. Overall, in this study the authors provided evidence that apigenin can sensitize SEC models to 5-FU cytotoxicity and offer new insights for targeted cancer therapy.

#### Human leukemia cell

In another study carried out by Mahbub et al., treatment of human leukemia cell lines including TIB-152, TIB- 202, CCL-119, and CCL-243 cell lines by 5-FU alone demonstrated a significant decrease in ATP levels in all of the cell lines. When 5-FU was concurrently used with apigenin, it resulted in a synergistic depletion in ATP levels. This combination therapy also induced cell cycle seizure and apoptosis in leukemia cell lines. 5-FU significantly enhanced the activity of caspase 3 in all lymphoid and myeloid leukemia cell lines. Its co-administration with apigenin exhibited synergism compared with monotherapy [6]. Nevertheless, the study lacked the consideration of exact molecular mechanisms and cell signaling pathways of the combination therapy.

### Cetuximab

Cetuximab is a chimeric human-murine monoclonal antibody that targets the epidermal growth factor receptor and has been used against various cancers in clinical trials [59, 60]. However, like other chemo drugs, it may be allied with treatment-related toxicity. Hence, co-therapy with another drug would be helpful to improve its efficacy and safety.

#### Head and neck squamous cell carcinoma

Complementary benefits of cetuximab and apigenin were investigated in three separate studies [61, 62], using this regimen to overcome the cetuximab resistance in HNSCC. Boeckx et al. considered the cytotoxic effect of the apigenin as an ERK1/2 inhibitor in the cetuximab-resistant cell lines including Cal27 and LICRHN1. They treated both cell lines with apigenin alone and calculated mean values of IC_50_, 22.22 µM for Cal27 and 34.32 µM for the LICRHN1 cell line. Afterwards, they evaluated the cytotoxic effect of co-administration of apigenin and cetuximab. They observed a significant reduction in cell survival during combined treatment of Cal27 and LICR-HN1 cell lines compared to the drugs monotherapy. On the other hand, the results of this study, represented that rat sarcoma/mitogen-activated protein kinase (RAS–MAPK) pathway overexpression has a critical role in the cetuximab resistance in Cal27 and LICR-HN1 cells. Although the authors concluded that the inhibition of the RAS–MAPK signaling pathway by apigenin as an ERK1/2 inhibitor could be applied as a new therapeutic strategy to overcome the cetuximab resistance in HNSCC [61], the exact molecular mechanism of this claim was not investigated in this study.

Secondly, the same authors demonstrated that the combination therapy by apigenin and cetuximab causing notable suppression of cystatin E/M (CST6), FOS-like antigen 1 (FOSL1), plasminogen activator urokinase (PLAU), and vimentin (VIM) expression in the LICR-HN5 R9.1 cells [63]. Furthermore, S100A8 expression was increased in both LICR-HN5 R9.1 and SC263 10.2 cells after combination therapy compared to the treatment by cetuximab alone, and contrary to LICR-HN5 R9.1 cells, the expression of PLAU was enhanced in the SC263 R10.2 cells after co-treatment by apigenin and cetuximab [63]. A transcription factor; activator protein-1 (AP-1) controls all genes of various events such as cell growth, apoptosis, cell differentiation, and proliferation [62]. Keeping the regulatory functions of AP-1 in account for cancer cells, it seems that targeting AP-1 may be a potential therapeutic strategy against different cancers [64]. It has been reported that CST6 down-regulation was observed in metastatic primary cancer cells and loss of its function has a vital role in the progression of various types of cancers including breast cancer, glioma, and lung cancer [65]. Therefore, it seems rational that an increase in CST6 expression may be effective in cancer prevention, however in the study carried out by Boeckx et al. [63], its reduction was intensified by co-treatment with cetuximab and apigenin. Hence, such paradoxical findings are seemingly inadequate for cetuximab-apigenin co-therapy and more similar studies are required to clarify the exact cell signaling pathway. FOSL1 is a leucine zipper transcription factor that is involved in the formation of the AP-1 transcription factor complex [66]. In a study performed in 2016 [67] (after Boeckx's study), the role of FOSL1 in HNSCC cells growth and resistance to the chemotherapy was highlighted. The silencing of the *FOSL1 gene* in HNSCC cells (FaDu cell line) resulted in inhibition of the cell growth and migration in HNSCC cells [67]. On the other hand, there is strong evidence supporting the role of PLAU in HNSCC. Overexpression of PLAU and its receptor induces tumor cells migration, invasion and consequently metastasis [68]. VIM is over-expressed when cancer cells undergo the epithelial–mesenchymal transition (EMT) and it plays a key role in the invasive behavior of tumor cells [69]. Therefore, the down-regulation of FOSL1, PLAU, and VIM by the co-administration of cetuximab and apigenin may be considered as an innovative therapeutic strategy in the treatment and prevention of HNSCC in the future.

#### Human nasopharyngeal carcinoma

In 2018, Hu et al. [70] combined apigenin with cetuximab to investigate its anti-tumor activity on the human nasopharyngeal carcinoma (NPC) in vitro and in vivo. According to the obtained results, simultaneous administration of apigenin and cetuximab decreased the viability and growth of HONE1 and CNE2 cells more than when apigenin or cetuximab was used alone. Apigenin also improved the percentage of apoptosis and cell cycle arrest when it was used in combination with cetuximab. Besides, apigenin amplified the inhibitory effects of cetuximab on the EGFR signaling pathway. The expression of down-stream proteins of the EGFR pathway including p-EGFR, p-AKT, p-STAT3 and Cyclin D1 was suppressed in apigenin and cetuximab groups compared to the control groups or when apigenin or cetuximab was used alone in both HONE1 and CNE2 cells. In the NPC nude mice simultaneously treated with apigenin and cetuximab, the tumor size surprisingly reduced compared to the control group and those treated with apigenin or cetuximab alone [70]. EGFR; as a signal transducer, has imperative regulatory roles in different cellular events such as cell differentiation, proliferation, and survival and is over-expressed approximately in 80% of NPCs [71]. It has been stated that the overexpression of EGFR is associated with resistance to chemotherapy, poor prognosis, and a more aggressive phenotype of cancers [72]. Therefore, it seems that targeting EGFR signaling pathway by cetuximab and amplification of its anti-tumor activity by apigenin may be considered as a potential treatment for NPC.

### Cisplatin

Cisplatin (cis-diammineplatinum (II) dichloride) is a commonly used chemo drug for the treatment of various types of human malignancies; therefore it is also entitled “cancer penicillin” [73]. Nonetheless, due to drastic undesirable effects in normal tissues including nephrotoxicity, neurotoxicity, ototoxicity, and emetogenicity, its chemotherapeutic usage is limited [74]. Among the side effects of cisplatin, nephrotoxicity is the major concern for cancer patients [73]. Owing to the significance of cisplatin in cancer treatment, many investigations have focused on protective strategies to minimize cisplatin side effects especially nephrotoxicity [75]. Chemoprotection with flavonoids like apigenin is one of the possible solutions to minimize the adverse effects of cisplatin.

**Fig. 2:**
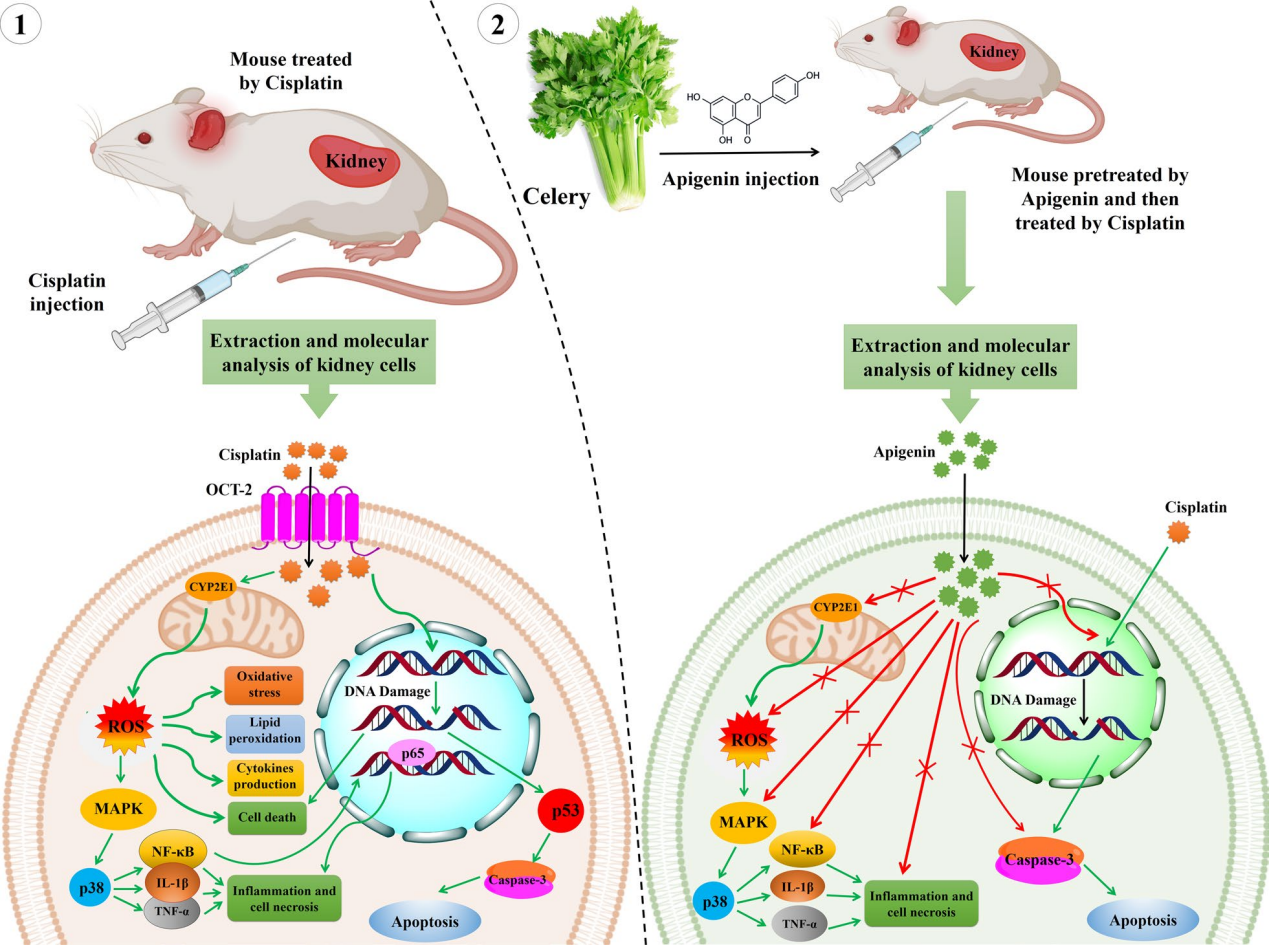
Chemoprotective effects of apigenin. Part. 1, a mouse was administrated with cisplatin. In order to investigate the molecular mechanisms of renal injury by cisplatin, followed by treatment, kidney cells were extracted and molecular analysis were performed. Cisplatin causes nephrotoxicity by oxidative stress, inflammation, apoptosis, and necrosis. Cisplatin enters into the renal cells by OCT-2 and over-activates CYP2E1 as an active producer of ROS in mitochondria and triggers ROS production which in turn leads to oxidative stress, lipid peroxidation, different cytokines (IL-6 and IL-1β) production, MAPK pathway activation and cell death. CYP2E1 plays a pivotal role in the promotion of oxidative stress in the kidney and increases cisplatin-induced nephrotoxicity. The produced ROSs by CYP2E1 can activate NF-κB and MAPK. NF-κB is a pro-inflammatory transcription factor and regulates the expression of different inflammatory factors. NF-κB is separated in the cell cytosol by binding to an inhibitory protein, IκB. Whenever, NF-κB is stimulated by stimuli such as viral, bacterial or other pathogens, a proteasome ubiquitinates and degrades IκB and releases NF-κB to translocate to the nucleus. In the nucleus it triggers the expression of target genes, like TNF-α, IL-1β and TGFβ which play important roles in cisplatin-induced kidney injury. Part 2, a pre-treated mouse by apigenin, was treated with cisplatin. The molecular analysis of the renal cells demonstrates that pre-treatment by apigenin significantly reduced cisplatin-induced renal injury by anti-oxidant and anti-inflammatory effects. Apigenin significantly suppressed the cisplatin-induced increase in the CYP2E1 levels in the mouse. Subsequently, it inhibited the renal oxidative stress, lipid peroxidation, generation of pro-inflammatory cytokines like TNF-α, IL-1β and TGFβ from the kidney tissue of cisplatin-treated mouse. Apigenin protected kidney cells against DNA damage (apoptosis) after cisplatin administration. It also significantly decreased the activities of NF-κB p65 and p38MAPK that were increased by cisplatin. (Inhibition  Activation)

The nephrotoxicity induced by cisplatin is associated with ROS production and p53 activation [76]. It has also been demonstrated that apigenin selectively affects apoptosis induction and cell growth inhibition in cancerous cells without influencing normal cells [77]. In this regard, Ju et al. [78] examined the effects of combination therapy on the complications posed by cisplatin in human renal proximal tubular epithelial (HK-2) cells. Pretreated HK- 2 cells with different concentrations of apigenin (5–20 μM) were treated with 40 μM cisplatin for 12 and 24 h. They found that induced apoptosis by cisplatin in HK-2 cells was suppressed by apigenin. Apigenin also reduced: (1) the cisplatin-induced caspase-3 activity, (2) the cleavage of caspase-3 substrate (PARP), and (3) the cisplatin-induced phosphorylation and expression of p53 in HK-2 cells. The results of this study indicated that apigenin could render chemoprotection against cisplatin on HK-2 cells by inducing PI3K/AKT pathway. To investigate the cisplatin-sensitizing effects of apigenin in a p53-dependent manner, Liu et al. [79] co-treated different human cancer cell lines with apigenin and cisplatin. Apigenin co-administration with cisplatin, enhanced cisplatin-induced apoptosis via the up-regulation of p53, decreased cell proliferation, increased MAPK, and pro-apoptotic proteins activation. According to the obtained results from two aforementioned studies [78, 79], apigenin chemoprotects and chemosensitizes by inactivation of p53, however this remarkable molecular behavior of apigenin in normal and cancerous cells require more investigations. In the other study [80] chemoprotective effect of apigenin against cisplatin-induced renal dysfunction was investigated in vivo. The male BALB/c mice were divided into six groups including the cisplatin group (20 mg/kg), cisplatin plus apigenin group (5, 10 and 20 mg/kg), and apigenin group (20 mg/kg). According to the results, pre-administration of apigenin markedly reduced renal destruction owing to the dramatic reduction in the levels of serum creatinine, BUN, GSH-PX, and SOD compared to the cisplatin-treated group. The levels of inflammatory cytokines including TNF-α, IL-1β, and TGF-β were decreased in the group which was simultaneously treated by cisplatin and apigenin compared with the cisplatin-treated group in the renal samples. In addition, apigenin caused suppression of cytochrome P450 2E1 (CYP2E1), phosphorylated necrosis factor kappa B (p-NF-κB p65), and p-p38 MAPK in cisplatin-induced kidney damage. CYP2E1 actively produces ROS [81] and increases cisplatin-induced oxidative stress in the kidney. NF-κB, as a pro-inflammatory transcription factor, is inhibited by the inhibitory IκB protein in the cell cytoplasm. External stimuli like viruses or bacteria cause the ubiquitination of IκB and NF-κB release. Then, NF-κB enters the nucleus followed by transcription of target genes, such as TNF-α, IL-1β and TGFβ to promote inflammatory responses. These proteins play a key role in the nephrotoxicity induced by cisplatin. These results demonstrated that the pretreatment of mice by apigenin dramatically reduced cisplatin-induced kidney dysfunction and renal injury due to its anti-oxidant and anti-inflammatory properties [80]. Figure 2 summarizes the chemoprotective effects of apigenin. In a similar study, the molecular mechanism of reno-protective effects of apigenin were investigated in vivo on 8 groups of adult female Wistar Albino mice. According to the results, apigenin strikingly reduced blood BUN, serum creatinine, caspase-3, TNF-α, and IL-6. Moreover, histopathological cisplatin-induced kidney injury was improved by apigenin administration. It seems that apigenin being an anti-oxidant and anti-inflammatory substance might possess nephron-protective effects [82].

#### Melanoma cells

Primarily, in a study conducted in 2000 by Caltagirone et al. [83] the effects of apigenin on the growth and metastatic behavior of B16-BL6 melanoma cells were investigated in vivo. For this purpose, apigenin (25 mg/kg), cisplatin (2 mg/kg), and their combination (25 mg/kg of apigenin plus 2 mg/kg of cisplatin) were intravenously injected into 4 groups, (control, apigenin, cisplatin, and combination therapy groups) 3 days after tumor cell injection. The obtained results showed that apigenin and cisplatin alone reduced the tumor volume but their co-administration turned to be more effective than cisplatin or apigenin alone. However, the authors did not focus on the molecular mechanisms underlying single or combinatorial therapy by the above-mentioned anti-cancer drugs [83].

#### Laryngeal carcinoma

Hypoxia is a condition associated with cancerous cells which causes an increase in glucose uptake and metabolism. Glucose transporter-1 (GLUT-1); a hypoxic marker, has a key role in malignant glucose metabolism. Over-expression of GLUT-1 is linked with the activation of PI3K/AKT signaling pathway, resulting in the GLUT-1 expression. Recently, GLUT-1 and PI3K/AKT have been identified to be closely involved in resistance to the chemotherapy of some human cancers. Xu et al. [84] supposed that GLUT-1 over-expression and AKT hyper-phosphorylation could be associated with the resistance of laryngeal carcinoma Hep-2 cells to cisplatin. Afterwards, they investigated the apigenin chemosensitizing effects on cisplatin considering the levels of GLUT-1 and p-AKT in Hep-2 cells. The results suggested that apigenin could significantly increase the cisplatin-induced inhibition of Hep-2 cells growth and reduce the expression of GLUT-1 mRNA, GLUT-1, and p-AKT proteins in Hep-2 cells in a dose and time-dependent manner during the co-administration with cisplatin. Conclusively, the over-expression of GLUT-1 and hyper-phosphorylation of AKT may be associated with the insensibility of laryngeal carcinoma Hep-2 cells to cisplatin. Therefore, apigenin could decrease the resistance to cisplatin by the suppression of GLUT-1 and p-AKT expression [84].

#### Breast cancer

The effect of the co-treatment of cisplatin and apigenin was probed on the two triple-negative breast cancer (TNBC) cell lines including MDA-MB-231 and HCC1806. The study suggested that the co-therapy suppressed the activity of the telomerase; its over-expression is one of the cell death escaping strategies in cancerous cells. Telomerase is a complex protein comprising of hTERT, Hsp90, p23, and other proteins. Therefore, the subdual of these proteins by the co-administration of apigenin and cisplatin could be considered as a favorable strategy in breast cancer treatment [85].

#### Prostate cancer

The evaluation of the combination therapy of apigenin (15 mM) plus cisplatin (7.5 mM) on CD44+ prostate cancer stem cell demonstrated a noteworthy decrease in p-PI3K, p-Akt, and NF-kB protein levels. Co-administration of apigenin and cisplatin halted the cell cycle by up-regulation of p21, cyclin-dependent kinases-2, 4, and 6 (CDK-2, 4 and 6) [86].

#### Human ovarian cancer

**Fig. 3:**
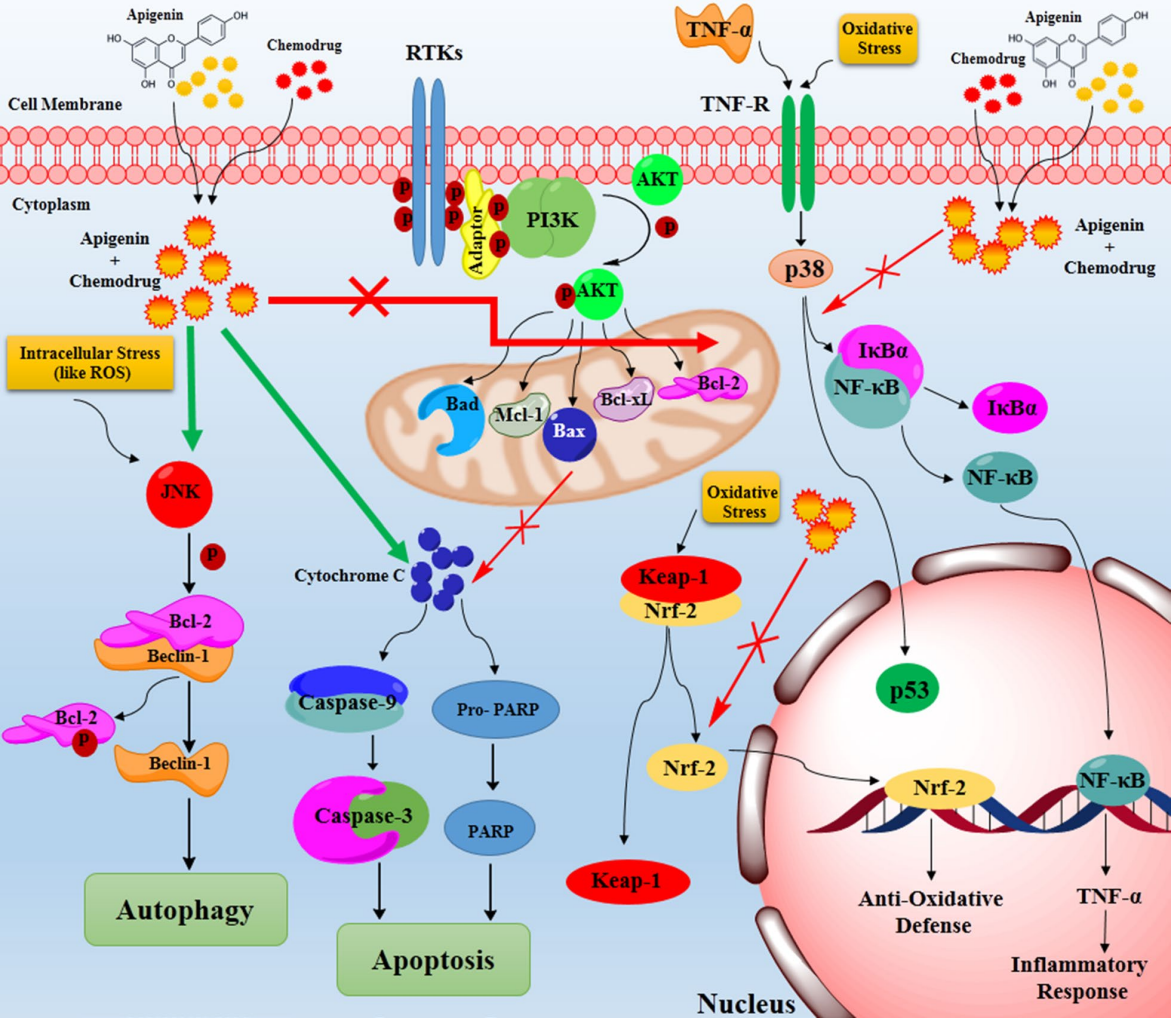
The schematic apoptosis and autophagy induction by apigenin when it is simultaneously used with a chemo drug. Two dimers of the RTK phosphorylate together in multiple tyrosine sites within the RTK intracellular domain which mediate different downstream signaling cascades such as PI3K/AKT pathway. PI3K/AKT signaling is initiated through interaction between activated RTK and adaptor proteins. PI3K phosphorylates AKT protein and p-AKT triggers the activation of several proteins playing critical role in apoptosis. The over-activation of PI3K/AKT results in the over-expression of the anti-apoptic proteins such as Bcl-2, Bcl-xL, Bax, Bad and Mcl-1. These proteins inhibit the down-stream cascade of apoptosis and cause indefinite cell proliferation. Co-administration of apigenin and chemo drugs induces apoptosis by the inhibition of the anti-apoptic proteins. Autophagy is the other activated mechanism by apigenin-chemo drugs suggested in different studies. In autophagy, apigenin-chemo drugs increases JNK. It has been reported that activation of JNK can induce Bcl-2 phosphorylation, resulting in the release of Beclin-1and autophagy activation. Generally, these results revealed a fundamental role of JNK in apigenin-induced autophagy and apoptosis. Nrf2, a redox-sensitive transcription factor, regulates the expression of cytoprotective genes and protective cells against oxidative/electrophilic agents-induced damages. Nrf2 binds to the AREs in the promoters of various cytoprotective genes and regulates their expression. Nrf2 overexpression increases chemoresistance, therefore, its inhibition by apigenin plus chemo drugs sensitizes different cancer cells against chemo drugs. NF-κB, is inhibited by the inhibitory IκB protein in the cell cytoplasm. External stimuli causes to the ubiquitination of IκB and NF-κB release. Then, NF-κB enters to the nucleus and starts the transcription of target genes, such as TNF-α, IL-1β and TGFβ to promote inflammatory responses. These proteins play critical roles in the nephrotoxicity which is induced by some of the chemo drugs such as cisplatin. Apigenin dramatically reduced chemo drugs-induced kidney dysfunction by anti-oxidant and anti-inflammatory effects by inhibition of the NF-κB- IκB complex separation by p38 protein. (Inhibition  Activation)

Intriguingly, apigenin can augment the inhibitory effects of cisplatin on the proliferation of human ovarian cancer cell lines (SKOV3 and cisplatin-resistant SKOV3/DDP). The combination of apigenin and cisplatin markedly induced apoptosis and down-regulated cyclin D, cyclin B, and cyclin E compared to the cells treated by each agent alone. The astounding chemosensitizing effect of apigenin on SKOV3/DDP cells made them vulnerable to cisplatin even more than SKOV3 cells. Moreover, apigenin induced the caspase-3 (as an apoptosis inducer) cleavage and decreased Bcl-2 (as an anti-apoptotic factor) in both cell types. Apigenin significantly reduced Mcl-1, (which belongs to an anti-apoptotic proteins family (Bcl-2 family) and plays an important role in the apoptosis inhibition) at mRNA and protein level, both in SKOV3 and SKOV3/DDP cell lines [87, 88]. It has been suggested that this mechanism could be responsible for apigenin cytotoxic and chemosensitizing effects in human ovarian cancer [87, 89] (Fig. 3).

### Cyclophosphamide (CYCLO)

Cyclophosphamide (CYCLO) was approved in 1959 by FDA. According to the American Society of Health-System Pharmacists, on 2 January 2017, it has been used for the treatment of various type of cancers including lymphoma, multiple myeloma, leukemia, ovarian cancer, breast cancer, small cell lung cancer, neuroblastoma, and sarcoma.

#### Secondary acute myeloid leukemia

Cyclophosphamide-induced mutations may result in secondary acute myeloid leukemia which is called therapy-related AML [90]. In two different studies, the protective effects of apigenin on the mutagenic and genotoxicity effects of CYCLO were considered. Boculic et al. selected *Salmonella typhimurium* for in vitro mutagenesis assay using 400 µg/plate and 800 µg/plate of apigenin. Although, apigenin did not inhibit the CYCLO-induced mutations, the results demonstrated that apigenin significantly reduced CYCLO-induced genotoxic damage in vivo [91]. In a cell culture-based study, apigenin synergistically increased the sensitivity of lymphoid and myeloid leukaemia cells to the CYCLO by decreasing ATP and glutathione levels and increasing the activity of caspase 3, caspase 8, and caspase 9. According to the research findings, apigenin caused the accumulation of lymphoid leukaemia cells in the S phase of the cell cycle when combined with CYCLO. Furthermore, the cell cycle in myeloid cells was arrested in G2/M and/or S phase when apigenin was co-administrated with CYCLO. Combined treatment by apigenin and CYCLO increased in γH2AX foci in all examined cell lines, showing DNA damage owing to the apoptosis by this co-therapy [92].

### Doxorubicin

#### Gastric cancer

Doxorubicin (DOX) was primarily used as a chemo drug against gastric cancer. Prolonged treatment with doxorubicin in gastric cancer patients results in the development of drug resistance and tumor regression [93]. In 2011, the mutagenic and anti-genotoxic effects of apigenin and DOX were studied in vitro (400 µg/plate of apigenin + 0.2 µg/plate of DOX) and in vivo (100 mg/kg of apigenin + 5 mg/kg of DOX) [91]. Apigenin reduced doxorubicin-induced mutagenicity in vitro, however, no significant reduction was observed in micronucleus frequency in the animal study. The authors attributed this finding to the shortcomings in the metabolic transformation of the drugs at the used doses [91].

#### Hepatocellular carcinoma

In 2013, Gao et al. [94] substantiated the chemosensitizing effects of apigenin on hepatocellular carcinoma cell lines (BEL-7402/ADM) to DOX. Apigenin halted the cell cycle in the S-phase when it was co-administered with DOX. Fluorometric analysis corroborated that apigenin caused intracellular accumulation of DOX, hence, chemosensitizing cancerous cells against DOX. Consequently, the authors found that apigenin (10 µM) significantly enhanced the cytotoxicity of DOX (10 µM) in BEL-7402/ADM cells. The molecular investigation demonstrated that apigenin exerted its chemosensitizing effects on DOX by inhibiting Nrf2 both in transcription and translation [94].

In two other separate studies performed by the same authors on the same cell line, apigenin markedly enhanced BEL-7402/ADM cells sensitivity to DOX by an increase in miR-101 [95] and miR-520b [96] expressions. The miR-101 sensitizes BEL-7402/ADM cells to the chemo drug by targeting the 3′-UTR of Nrf2, finally silencing and down-regulating Nrf2 [95]. The Nrf2; a redox-sensitive transcription factor, regulates the expression of cytoprotective genes, hence, protecting cells against oxidative/electrophilic agents-induced damages. As a transcription factor, Nrf2 binds to the anti-oxidant-response elements (AREs) in the promoters of various cytoprotective genes such as heme oxygenase-1 (HO-1), NAD(P)H quinone oxidoreductase 1, aldo–keto reductases, and several adenosine triphosphate-dependent drug efflux pumps and regulates their expression [97]. Recently, Nrf2 has drawn the researchers' attention as a potential pharmacological target to overcome chemoresistance owing to the reports that Nrf2 overexpression increases chemoresistance, while its inhibition sensitizes different cancer cells against chemo drugs [98, 99] (Fig. 3).

A study revealed that the co-administration of DOX and apigenin dwindled the expression of hexokinase 2 (HK2) and lactate dehydrogenase A (LDHA) in the human hepatocellular carcinoma cell line, HepG2. HK2 and LDHA are the glycolytic pathway genes playing crucial roles in the Warburg effect in cancer cells and their inhibition by DOX and apigenin co-administration possibly hamper the growth and proliferation of cancer cells [100].

#### Prostate cancer

To investigate whether apigenin could sensitize resistant prostate cancer cells to DOX, TaxR cells were administered with 20 nM DOX alone or in the combination with 10 mM apigenin. The combination of DOX and apigenin reduced TaxR cells growth to 50% compared to the DOX or apigenin alone. After the co-treatment of TaxR cells, the authors found that apigenin resensitized DOX-resistant prostate cancer cells by impeding ABCB1 expression [101]. Recently, it has been reported that ABCB1 corresponds to trigger the development of chemo drug resistance [102], thus, it seems that ABCB1 down-regulation could be an effective strategy in sensitizing cancer cells to different chemotherapies.

#### Uterine sarcoma

Co-administration of 2 and 8 µM DOX with 10 µM apigenin promoted significant intracellular accumulation of DOX in Human DOX-resistant uterine sarcoma cells (MES-SA/Dx5) compared to DOX alone. This combination therapy curtailed cellular GSH levels by ABCB1 inhibition, therefore, apigenin as a potential adjuvant to anti-cancer treatment may overcome ABCB1-mediated drug resistance in cancer [103].

#### Breast cancer

The study carried out by Seo et al., suggests that apigenin can reduce the expression of ABCB1 in human DOX-resistance breast cancer cell line (MCF-7/ADR) in the presence of DOX. Indeed, apigenin-induced drug resistance has been ascribed to the inhibition of the STAT3 pathway which promotes cell growth and proliferation [104].

#### Human leukemia

The studies indicate that the combined treatment of apigenin and DOX can reduce ATP levels in human leukemia cells due to the cell toxicity and DNA damage. It also boosts caspase-3, 8, and 9 activities and seizes the cell cycle in S and G2/M phases in human leukemia cells [105]. In two separate animal studies [106, 107], DOX-induced cardiac injury, cardiomyocyte apoptosis, and autophagy in mice were overcome by apigenin administration. Apigenin might exert its cardio-protective effects by activation of the PI3K/AKT/mTOR signal transduction pathway crucial for the cell apoptosis and autophagy [106]. Moreover, a remarkable diminution in apoptotic proteins including caspase 3 and Bax, and augmentation in anti-apoptotic protein like Bcl2 was observed in DOX plus apigenin administered group [107].

#### Human ovarian adenocarcinoma

Human ovarian adenocarcinoma, SKOV-3 cells were chosen to investigate DOX-sensitizing effects of apigenin and doxorubicin. This simultaneous treatment restricted the growth of SKOV-3 cells in a dose and time-dependent manner. Apigenin induced early apoptosis, improved caspase 9 activity, enhanced Bcl-2 and COX-2 expression, and arrested the cell cycle at the G2/M phase within 24 h during co-treatment with DOX, compared to the DOX treatment alone. It seems that apigenin could be an alternative for sensitizing SKOV-3 cells to DOX to rapidly induce early apoptosis [108].

### Gemcitabine

Gemcitabine was initially used as an anti-viral drug, but preclinical experiments proved it toxic for leukemia cells in vitro [109]. In the 1990s, based on clinical trials, gemcitabine significantly prolonged the survival rate of patients with pancreatic cancer, therefore, it received the FDA approval in 1996, 1998, and 2004 to treat pancreatic cancers, non-small cell lung cancer, and metastatic breast cancer, respectively [110]. However, its monotherapy turned to be ineffective to control mortality, therefore its co-treatment with apigenin can be considered to ameliorate its efficacy.

#### Human pancreatic cancer

In a study, apigenin (13 µM) boosted the anti-proliferative effects of gemcitabine (10 µM) on BxPC-3 human pancreatic cancer cells, but the authors did not explain potential cell signaling pathways underlying simultaneous administration of apigenin plus gemcitabine [52]. Lee et al., demonstrated that combination treatment of gemcitabine and apigenin caused tumor shrinking in the xenograft model of pancreatic cancer cells, while single treatment of gemcitabine inhibited growth and induced apoptosis. Gemcitabine-induced activity of AKT and NFκB was suppressed during the co-administration of gemcitabine with apigenin [111]. Apigenin in combination with gemcitabine suppressed CD18 and AsPC-1 pancreatic cancer cells proliferation, more than gemcitabine alone. Combination therapy seized the cell cycle within S and G2/M phase and enhanced apoptosis in the cancerous cells compared to the single therapies. The inhibition of AKT and NFκB pathway by apigenin might be an important molecular mechanism to potentiate gemcitabine- induced anti-tumor activity in pancreatic carcinoma [112].

### Paclitaxel

Paclitaxel (PTX) is an anti-cancer drug that is widely administered in the treatment of human breast, ovarian, lung, cervical and pancreatic cancers [113]. The predominant therapeutic concern in oral administration is its low bioavailability due to the poor solubility and first-pass metabolism occurring in the liver and small intestine. This event has been attributed to the PTX metabolism by enzymes or ABCB1 in the intestinal cells. To improve the efficiency of oral delivery of PTX, some investigations have been performed by the suppression of ABCB1 and metabolic enzymes. PTX is a substrate of ABCB1 and several studies have reported that its oral bioavailability was substantially increased by PTX co-administration with ABCB1 inhibitors [114]. As formerly mentioned, apigenin sensitized prostate [101], uterine [103], and breast [104] cancer cells to DOX by ABCB1 inhibition. Choi et al. investigated the effect of apigenin on the bioavailability of PTX in the animal model. After oral administration of rats by 40 mg/kg PTX plus 0.4 mg/kg apigenin, they noticed distinct inhibition of ABCB1 activity compared to the group which was treated only by PTX. Apigenin also significantly increased the terminal half-life of PTX when it was used orally. Taken together, the improvement of oral bioavailability of PTX by apigenin might be due to enhanced intestinal absorption owing to ABCB1 inhibition by apigenin [114, 115]. Xu et al. showed that apigenin could sensitize different human cancer cells such as cervical epithelial carcinoma, lung epithelial carcinoma, and hepatocyte carcinoma cells to PTX through inducing apoptosis by suppressing SOD activity leading to accumulation of ROS and caspase-2 cleavage [116].

The efficiency of combination therapy with apigenin plus PLX was further scrutinized on HepG2 cells and xenograft animal models. The administration of apigenin with PTX could constrain the expression of HIF-1α in hypoxic tumors by blocking p-AKT and HSP90 [117]. It has been stated that apigenin is a natural inhibitor of HIF-1α that suppresses HIF-1α expression via multiple mechanisms and reverses the hypoxia-induced resistance in solid tumor cells [117]. To reduce PTX concentration and subsequently enhance its effectiveness on SKOV-3 cells, PTX plus graphene oxide coated nanotised apigenin (GO-NA) was considered as a combination therapy. The results of the study revealed that GO-NA plus PTX synergistically inhibited cells proliferation contrary to GO-NA and PTX alone. GO-NA plus PTX markedly hampered SOD activity, caused ROS accumulation, halted cell cycle, up-regulated caspase-3 and Bax, and down-regulated of Bcl-2, representing apoptosis [118].

### Sorafenib

Sorafenib (also called Nexavar); a kind of kinase inhibitor, was approved for the treatment of advanced kidney malignancy in 2005. Then in November 2007, FDA approved sorafenib for use in patients with inoperable HCC [119]. However, sorafenib resistance occurs in most HCC patients after 6 months of the treatment. To overcome this limitation, the effects of apigenin on reversing sorafenib-induced resistance were investigated in HCC cells (HepG2). Cytotoxic effects of sorafenib on HepG2 cells were intensified when it was used with apigenin. Furthermore, the combination therapy including apigenin plus sorafenib was associated with escalation in the percentage of apoptotic cells, reduction in cell migration, and cell cycle halt compared to the single therapies. Simultaneous treatment of HepG2 cells, with 50 µM apigenin and 5 µM sorafenib significantly enhanced the expression of apoptotic genes including caspase 3, caspase 8, caspase 10, BID, p21 and p16 as compared to their expressions in the single treatment groups [120]. These results demonstrated that the combination of apigenin and sorafenib has synergistic effects on HCC and might be considered as a promising strategy in the treatment of the inoperable HCCs.

### Tamoxifen

Tamoxifen (TMX), the chief chemotherapeutic agent, is used to treat patients with estrogen receptor (ER-α) positive breast cancer. Nonetheless, nowadays TMX resistance has become an important clinical problem and the underlying mechanisms of this issue are not completely clarified yet [121]. To cope up with this challenge, some studies have scrutinized the chemosensitizing effects of apigenin to TMX on breast cancer cells and xenograft animal models. In 2003, Samuel et al., induced breast cancer in female albino Wistar rats and treated them with apigenin (50, 100, 200 mg/kg) and TMX (50 mg/kg). They found that apigenin at 100 and 200 mg/kg doses had a maximal effect in boosting the activity of anti-oxidant enzymes (SOD, GPx, and CAT) and suppressing VEGF expression compared to the TMX alone [122]. In another study, the combination of apigenin with TMX had synergistic, growth-inhibitory effects on breast cancer cells. Furthermore, apigenin dwindled ER-α, AIB1, and multiple protein kinases (p38, PKA, MAPK, and AKT) expressions. AIB1; the ER-α co-activator, is often up-regulated in breast cancer, acts as an oncogene through transmitting kinase-mediating growth factor signaling to the ER-α, therefore, the inhibition of AIB-1 by simultaneous administration of apigenin and TMX could be effective for the treatment of TMX-resistance breast cancers [123].

### Abivertinib

Abivertinib is a novel tyrosine kinase inhibitor that can selectively target both mutant forms of EGFR and Bruton's tyrosine kinase (BTK). It has completed a registration trial for lung cancer and also has been administered to more than 600 patients worldwide, however, not yet been approved by FDA [124]. The most aggressive type of B-cell lymphoma (DLBCL) exhibited a poor prognosis to date. Tyrosine kinase inhibitors such as BTK inhibitors are attributed to the improvement of the patients' survival rates. To investigate the efficacy of apigenin and abivertinib on the inhibition of DLBCL progression, Huang et al., treated DLBCL cell lines (U2932, OCI-LY10) and xenograft animal model by apigenin, abivertinib, and apigenin plus abivertinib. According to the in vitro and in vivo findings, apigenin co-administration with abivertinib restricted cell proliferation and clone formation in DLBCL cells. The combination therapy-induced apoptosis (by suppression of BCL-XL expression and activation of caspase-3 and caspase-8), impeded cell proliferation (by down-regulation of the PI3K/mTOR pathway) and hampered the cell cycle within the G2/M phase. Furthermore, apigenin synergistically induced apoptosis when it was used with abivertinib as compared to the monotherapy [125].

### Apo2L-TRAIL

Apo2 ligand (Apo2L)/tumor necrosis factor-related apoptosis-inducing ligand (TRAIL), a member of the tumor necrosis factor family which binds to the death receptors (DR4 and DR5) as a cytokine and selectively promotes apoptosis in different cancer cells without any damage to normal cells. Recently, it has emerged as a promising anti-neoplastic agent and its recombinant form has been under clinical trials, however, like other chemo drugs the "drug resistance" is the predominant challenge in this case. Various malignancies are resistant to Apo2L/TRAIL [126] and Oishi et al., investigated that apigenin increased the Apo2L/TRAIL-induced apoptosis in human pancreatic cancer cells (DU145 and LNCaP cell lines). It could bind and inhibit adenine nucleotide translocase-2 (ANT2), causing increased Apo2L/TRAIL-induced apoptosis by up-regulation of DR5 [127]. The main finding of this study was to discover that ANT2 is the main target of apigenin.

### Chlorambucil (CLB)

Chlorambucil (CLB), an alkylating anti-cancer agent, is an important chemo drug that is used to treat human leukemia. Corresponding to the other alkylating agents, CLB causes DNA cross-links which in turn hinder DNA synthesis, induce cell cycle arrest and apoptosis. Many adverse effects such as nausea, vomiting, hair loss, nephrotoxicity, and immune-weakness are associated with CLB therapy in leukemia patients. To reduce CLB side effects, Mahbub et al., disclosed a synergistic reduction in ATP and GSH levels, an increase in cell cycle arrest (in G2/M and/or S phases), DNA damage (an increase in γH2AX foci), and apoptosis (through activation of caspase pathways) in human lymphoid and myeloid leukemia cells by the combination therapy of apigenin with CLB [92].

### Gefitinib (ZD1839, Iressa)

Gefitinib is an EGFR inhibitor recommended by clinical guidelines as a standard treatment for patients with advanced NSCLC [128]. However, some NSCLC patients are intrinsically resistant to the TKIs. Chen et al. suggested that co-administration of apigenin and gefitinib might sensitize the resistant NSCLC cells to the chemotherapy. They substantiated that in H1975 cells (harboring the mutant EGRF), the apigenin plus gefitinib inhibited cell growth, induced metastasis and cell cycle arrest within the G0/G1 phase, increased cleaved-caspase-3 and cleaved-PARP-1 expression, down-regulated Bcl-2 (as an anti-apoptotic protein), and up-regulated BIM and Bax (as pro-apoptotic proteins). Besides, the combination therapy resulted in a significant decrease in the phosphorylated levels of AMPK-α in H1975 cells in comparison with the administration of apigenin or gefitinib alone [129].

### Interferon gamma (IFN-γ)

Interferon-gamma (IFNγ), as a multifunctional cytokine, is generated by natural killer (NK) and T cells. It has vital roles in the innate and adaptive immune responses and recently has been applied for the treatment of a various cancers. The anti-neoplastic activity of IFNγ is based on its anti-proliferative, anti-angiogenic, and pro-apoptotic effects. Nonetheless, IFNγ may cause tumor cells activation and apoptosis inhibition. In vitro cell culture-based study showed that apigenin increased IFNγ-induced cytotoxicity by the amplified cell cycle arrest and apoptosis induction in HeLa cells. However, the exact molecular mechanism is yet to be known for this combination treatment [130].

### Methotrexate (MTX)

Methotrexate (MTX) is a chemotherapeutic agent that inhibits the di-hydrofolate reductase enzyme (DHFR). DHFR produces tetra-hydrofolate (THF); a critical cofactor in the synthesis of nucleotides, therefore, DHFR inhibition by MTX causes Depletion of THF resulting in cell death owing to suppress transcription and translation (DNA and RNA production). MTX is commonly used to treat several cancers including leukemia, breast, lung, and lymphoma cancers. High toxicity results in an incomplete treatment by MTX and lessens its potential effectiveness [131]. Interestingly, contrary to the previous reports in this article about the chemosensitizing role of apigenin, Mahbub et al. [6] demonstrated that apigenin was antagonized by MTX. When apigenin was combined with MTX, it increased ATP amounts in the Jurkat and THP-1 cell lines. This combination markedly reduced caspase 3 activity in both Jurkat and THP-1 cells. The molecular mechanisms of this antagonism are vague, but most probably they correlate with an elevation in GSH levels and reduction in DNA damage and apoptosis [6].

### Vincristine

Vincristine (also called leurocristine) is a chemo drug used to treat acute lymphoid and myeloid leukemia, Hodgkin's disease, neuroblastoma, and SCLC. Autophagy has a dual role in a cell, it either prolongs cell survival in scarcity of nutrients or results in cell death. Autophagy induction is usually connected with resistance to chemo drugs. In a study, TF1 cells (human erythroleukemic cell line) after treatment by apigenin, were exposed to vincristine. The results demonstrated a significant drop in the cytotoxic effect of vincristine compared to the control groups. To clarify the chemoprotective effects of apigenin against vincristine, apoptosis induction by the combinatorial treatment was analyzed. The number of apoptotic cells without any treatment, treatment with vincristine, and with apigenin were recorded as 10.7 ± 0.007%, 33.6 ± 1.4%, and 22.34 ± 0.4%, respectively. Intriguingly, vincristine treatment of the apigenin-pretreated cells showed 19.8 ± 0.2% apoptotic cells, a significant reduction in the number of Annexin-Vpositive cells compared to vincristine treatment alone. Therefore, it is obvious that apigenin protects TF1 cells against vincristine-induced cell death. Albeit, chemopreventive effects of apigenin could be generally an advantage, but such differential response may create resistance to the chemotherapy [132].

### Metformin

Metformin is an old anti-diabetic medicine and recently its anti-cancer effect has drawn researchers’ attention [133, 134]. In a recently published research, Warkad et al. [135], demonstrated that metformin abridged pancreatic cancer cells (AsPC-1 cells) viability by increasing ROS levels. They presented that it had a minimal cytotoxic effect on human primary dermal fibroblasts (HFD) as normal cells. According to their findings, metformin dwindled ATP production in mitochondria of HDF cells, but it did not change ATP levels in AsPC-1 cancer cells. As a result of the reduction in ATP levels, AMPK, p-AMPK, FOXO3a, p-FOXO3a, and subsequently manganese superoxide dismutase (MnSOD) levels were increased in HDF cells and an elevation in MnSOD levels reduced existing levels of ROS in normal cells, but not in cancer cells. Therefore, the increased levels of ROS in AsPc-1 cells was attributed to the MnSOD inactivation. Most chemo drugs induce ROS production and decrease ATP levels by binding with mitochondria and leading to mitochondrial damage. However, some agents such as metformin and apigenin make a mild leakage in the electron transport chain (ETC) without affecting cellular integrity and survival. Warkad et al. showed that normal fibroblasts viability was not affected after treatment with metformin (0.05 to 20 mM for 48 h) or apigenin (1 or 20 µM) alone, however, its combination with apigenin (0.05, 0.5 or 5 mM of metformin and 20 µM of apigenin) reduced mitochondrial membrane potential in HDF cells dramatically but did not affect cellular integrity and cell viability. They suggested that metformin and apigenin synergistically inhibited mitochondrial membrane potency and this effect was attributed to a notable increase in ROS levels in cancer cells. They assumed that the metformin-apigenin combination activated the ETC in mitochondria causing ROS production in cancer cells, which finally instigated irreversible DNA damage. This effect was not observed in the AsPC-1 cells treated by each drug alone. According to the findings, Warkad et al. concluded that cell growth inhibition and apoptosis induction by metformin-apigenin was cancer cell specific because they did not observe synergistic interaction between metformin and apigenin in HDF cells. In this study, an in vivo experiment on ASPC-1 xenograft demonstrated that the oral treatment with a lower amount of metformin (75 mg/kg) or apigenin (5 mg/kg) alone for 4 weeks did not affect the tumor size. However, concomitant administration of metformin and apigenin for 4 weeks synergistically reduced tumor volume. These results suggested that a combination of metformin and apigenin could be beneficial for examination in preclinical models of pancreatic cancer.

## Conclusion and future prospects

Combinatorial therapeutics are considered as requisite for effective cancer therapy. These combinatorial strategies aim to overcome drug resistance and augment anti-cancer properties. According to recent researches, combinatorial therapy with natural compounds such as apigenin can upsurge the anti-tumor effects and alleviate the side effects of chemo drugs. In a nutshell, the synergistic effect of apigenin and various chemo drugs on different cancer types revealed that apigenin boosts the effect of above-mentioned chemo drugs on cancer cell lines and xenograft models. Furthermore, it lessens their toxicity and resistance by down- or up-regulating the molecules in respective cell signaling pathways. Therefore, it seems that apigenin could be a potent chemosensitizer for these drugs. Clinical studies in this regard are still in infancy and according to the reported information in the clinical trials.gov, just one study has been documented about the anti-tumor activity of apigenin (NCT00609310). So, the authentication of anti-tumor activity of apigenin and its final approval still awaits more clinical trials [136, 137]. In addition, we lack the data for chemoprotective and chemosensitizing effects of this bioflavonoid in the clinical trial stage.

To subdue the limitations of apigenin bioavailability, researchers designed liposomal nanoparticles to enhance not only its bioavailability but also cancer cells sensitivity. Drug-loaded and dual drug-loaded liposomes possess substantial cytotoxic effects on cell lines contrary to the free drugs. The results proved that the highest cell cycle arrest and apoptosis activation was induced by dual drug-loaded liposomes. The cell signaling investigations indicated that the treatment by dual drug liposomes resulted in a significant up-regulation of 5' adenosine monophosphate-activated protein kinase (AMPK) [138]. The Warburg effect states that in varied oxygen conditions, cancer cells can increase their survival and thus cause to tumor aggressiveness. AMPK is a negative regulator of the Warburg effect maintaining cellular energy homeostasis. Therefore, the activation of AMPK could reverse the Warburg effect and cause ATP depletion-induced apoptosis [139, 140]. Cyclooxygenase-2 (COX-2) was significantly suppressed by dual drug liposome-activated AMPK in examined cancer cell lines. Moreover, a noteworthy reduction was also observed in hypoxia-inducible factor 1-alpha (HIF-1α) expression as a result of the synergistic activation of AMPK by dual drug loaded liposome treatment. HIF-1α, is a transcription factor that regulates different genes in critical events in cancer development, including angiogenesis, cell survival, glucose metabolism, and invasion. In human cancers, intra tumoral hypoxia causes overexpression of HIF-1α. Owing to the overexpression of COX-2 and HIF-1α in human cancers, their inhibition is considered to be the effective therapeutic strategy to target the angiogenesis and cell proliferation. Furthermore, ROS production was intensified by the dual drug- loaded liposomes in CRC cell lines. The designed nanocarriers were also tested on mice tumor xenograft model. Like the in vitro study, dual drug-loaded liposomes had greater anti-neoplastic and anti-tumorigenic effects [138, 141].

Apigenin can be a promising chemosensitizer and chemopreventive agent to minimize toxicity and intensify the effectiveness of current chemo drugs. According to the reviewed researches in this paper, there is a global consensus on apigenin usage as an adjutant in different combinatorial therapies and the most common mechanisms to amplify the chemo drugs’ efficacy are autophagy and apoptosis (Fig. 3). Besides, various mechanisms such as regulation of the cell cycle, inhibition of tumor cell migration, invasion, and stimulation of the immune response can be responsible for their sensitizing properties in co-therapies. Conclusively, we suggest future studies to deeply understand the effects and mode of action of apigenin in cancer therapy, particularly its bioavailability, chemosensitizing and chemoprotective properties.

Human clinical trials are a prerequisite to consider the potential usage of apigenin in the prevention and treatment of various cancers and to determine its optimal application conditions and doses. In addition to its significance in anti-cancer therapies, the health benefits of apigenin in humans are also not well-known due to a lack of research data. The possible reason for this may be its higher metabolic transformation and low bioavailability. Moreover, molecular mechanisms of apigenin can be considered in future studies. According to the information presented in clinicaltrials.gov, the pharmacological effects of apigenin are under phase 2 of the clinical study. They have scrutinized the anti-cancer effect of apigenin in clinical trials for colorectal cancer cells by Technische Universität Dresden. This review opens up a new horizon for research in chemotherapy. Further conducive research may revolutionize the current strategies to ameliorate the cancer therapy and facilitate widespread use of co-administered chemo drugs as modern medicines.

## Data Availability

Not applicable.

## Abbreviations

5-FU: 5-Fluorouracil
dTMP: Deoxythymidine monophosphate
dUMP: Deoxyuridine monophosphate
FDA: Food and drug administration
ErbB-2: Erythroblastic oncogene B-2
AKT: Protein kinase B
PI3K: Phosphoinositide 3-kinase
HNSCC: Head and neck squamous cell carcinoma
Bcl-2: B-cell lymphoma-2
BxPC-3: Pancreatic cancer cells
HCC: Hepatocellular carcinoma
ROS: Oxygen species
DΨm: Mitochondrial membrane
OM: Oral mucositis
SEC: Solid ehrlich carcinoma
Mcl-1: Cell leukemia 1
AMPK: Adenosine monophosphate-activated protein kinase
COX-2: Cyclooxygenase-2
HIF-1α: Hypoxia-inducible factor 1-alpha
EGFR: Epidermal growth factor receptor
RAS–MAPK: Rat sarcoma/mitogen-activated protein kinase
VIM: Vimentin
PLAU: Plasminogen activator urokinase
NPC: Nasopharyngeal carcinoma
STAT3: Signal transducer and activator of transcription 3
SOD: Superoxide dismutase
GPx: Glutathione peroxidase
CAT: Catalase
BUN: Blood urea nitrogen
GSH-PX: Glutathione peroxidase
Nrf2: Nuclear factor erythroid 2-related factor 2
ABCB1: ATP-binding cassette subfamily B member 1
AIB1: Amplified in breast cancer-1
OCT-2: Organic cation transporter 2
CYP2E1: Cytochrome P450 2E1
RTK: Receptor tyrosine kinases
Bad: BCL2 associated agonist of cell death
Bax: BCL2 associated X protein
Bcl-xL: B-cell lymphoma-extra-large
Nrf2: Nuclear factor erythroid 2-related factor 2
AREs: Antioxidant-response elements
NF-κB: Necrosis factor kappa B
TNF-α: Tumor necrosis factor alfa
IL-1β: Interleukin-1 beta
TGF-β: Tumor growth factor beta
